# Expression of GITR Enhances Multiple Myeloma Cell Sensitivity to Bortezomib

**DOI:** 10.1371/journal.pone.0127334

**Published:** 2015-05-14

**Authors:** Yinghao Zhao, Kun Zhang, Guangquan Li, Xingyi Zhang, Donglei Shi

**Affiliations:** 1 Department of Thoracic Surgery, The Second Hospital of Jilin University, Changchun, China; 2 Centralaboratory, The Second Hospital of Jilin University, Changchun, China; University of Oxford, UNITED KINGDOM

## Abstract

Recently tumor necrosis factor receptor super family member 18 (TNFRSF18, also called GITR) has been identified as a novel tumor suppressor gene in Multiple Myeloma (MM), undergoing aberrant DNA methylation-mediated gene expression silencing. Furthermore, the expression of GITR blocks canonical NF-κB activation in MM cells in response to TNFα. Bortezomib, a proteasome inhibitor, can induce NF-κB activation, which may significantly influence the drug response in MM patients. In this study, we aim to elucidate if GITR status is associated with response to Bortezomib in MM cells through regulating GITR mediated NF-κB blockade. We found that GITR was significantly downregulated in MM patients and cell lines. Overexpression of GITR inhibited non-canonical NF-κB activation induced by TNFα. Moreover, NF-κB inhibitor induced apoptosis in GITR-deficient MM cells in response to TNFα. In addition, overexpression of GITR could inhibit Bortezomib-induced NF-κB activation and enhance the cytotoxicity of Bortezomib in GITR-deficient MM cell line (MM1.S). In contrast, knockdown of GITR attenuated the cytotoxic effect of Bortezomib on GITR proficient MM (RPMI) cell line and increased NF-κB activation. Finally, overexpression of GITR enhanced the sensitivity to Bortezomib in co-culture with bone marrow stromal cells and significantly reduced the tumor growth in MM1.S xenograft mice. In conclusion, we demonstrated that GITR expression can enhance the sensitivity to Bortezomib by inhibiting Bortezomib-induced NF-κB activation.

## Introduction

Tumor Necrosis Factor receptor super family members (TNFRSFs) play an important role in the immune responses and inflammatory reactions [1–3]. One of TNFRSFs, TNFRSF18 (GITR), a recently identified novel tumor suppressor on chromosome 1p36, loss of which might be highly related to pathogenesis in differential human cancers [4–9]. It has been reported that GITR deficiency could result in increased cell proliferation and reduced apoptosis in human Multiple Myeloma (MM) [10]. NF-κB transcription factors play a key role in the survival and proliferation of many kinds of B-cell tumors, especially for multiple myeloma [11]. It has also been shown that mutations involved in the NF-κB pathway are present in 15–20% of MM tumors [12]. These mutations can lead to activation of the canonical and non-canonical NF-κB pathway [13]. Therefore, targeting the NF-κB pathway is an attractive therapy approach for MM [14]. In previous report, it has been shown that GITR expression also impacts the NF-κB activation in response to GITR ligand [10]. These findings above indicate that GITR might also be important to drug response through modulating NF-κB pathway since NF-κB inhibitors were developed to treat MM patients in the past years.

In this present study, we hypothesized that deregulation of GITR may play a pivotal role in modulating drug response in MM. Here, we showed that GITR is significantly downregulated in MM patients. Inhibition of NF-κB activity can significantly result in TNFα-induced apoptosis in GITR-deficient MM cell lines. In addition, expression of GITR correlates with Bortezomib sensitivity in MM cells, supported by overexpression and knockdown of GITR affecting the cytotoxicity of Bortezomib in MM cell lines. Furthermore, we also demonstrated overexpression of GITR impaired the interaction between MM cells and stromal cells and significantly decreased MM cell growth upon the treatment of Bortezomib. Finally, we showed that GITR expression could also enhance the effect of Bortezomib on inhibition of MM tumor growth in MM1.S xenograft mice model. These findings imply that GITR status is critical to response to Bortezomib in myeloma cells through regulating NF-κB pathway.

## Results

### GITR is downregulated in MM patients

**Fig 1:**
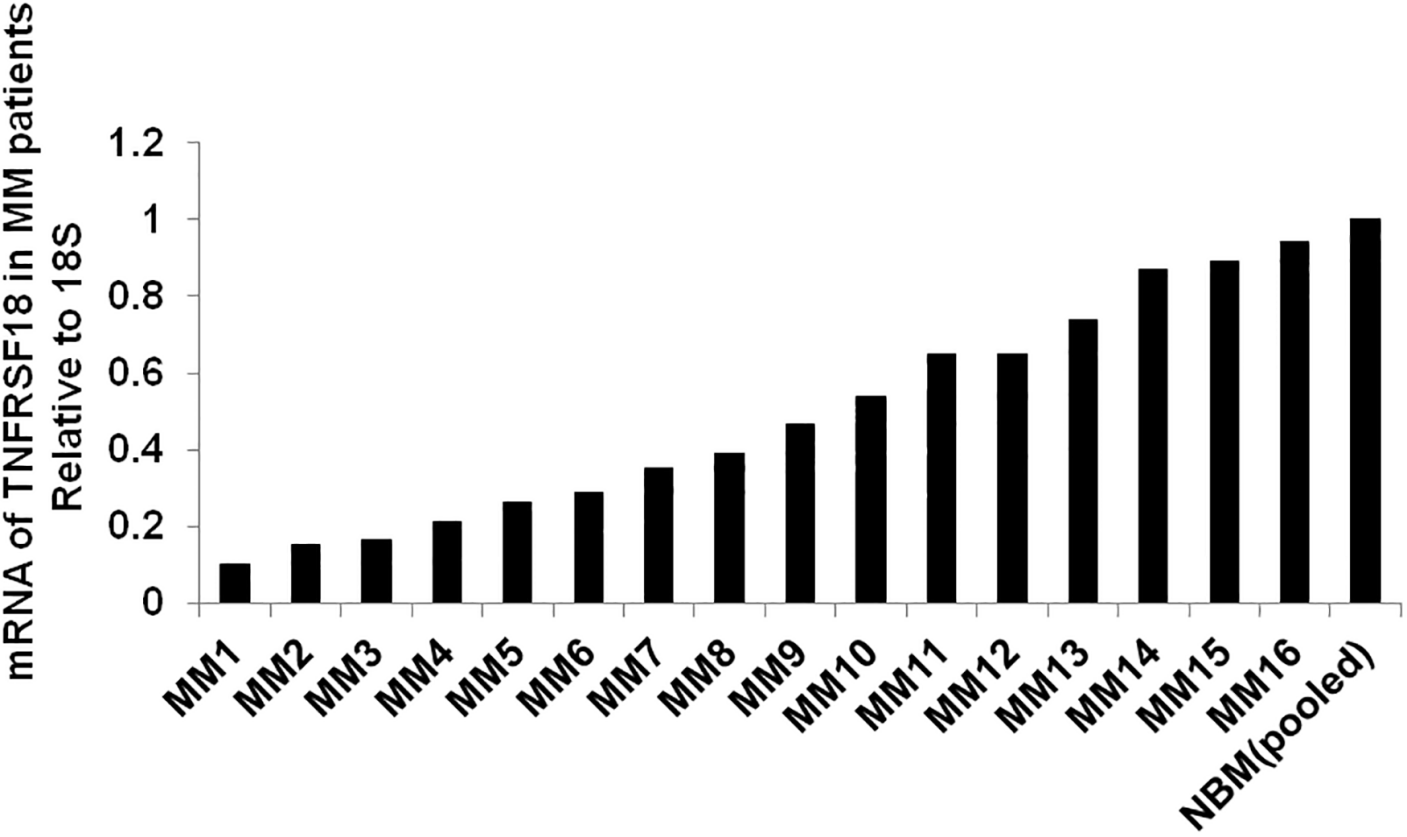
Expression of GITR in MM patients. mRNA of GITR from 16 MM patients and 20 pooled normal bone marrow specimens were assessed by real time-PCR. 18S was considered as the internal control.

First, using Real Time-PCR, we evaluated the expression of GITR in primary CD138^+^ bone marrow derived plasma cells from 16 MM patients by comparing to pooled normal bone marrow derived plasma cells (N = 20). We found that GITR levels of MM patients were significantly lower than the pooled normal groups (Fig 1), which is consistent with previous report [10]. These observations suggest that deregulation of GITR is very prevalent in MM.

### Overexpression of GITR inhibits TNFα-induced non-canonical NF-κB activation

**Fig 2:**
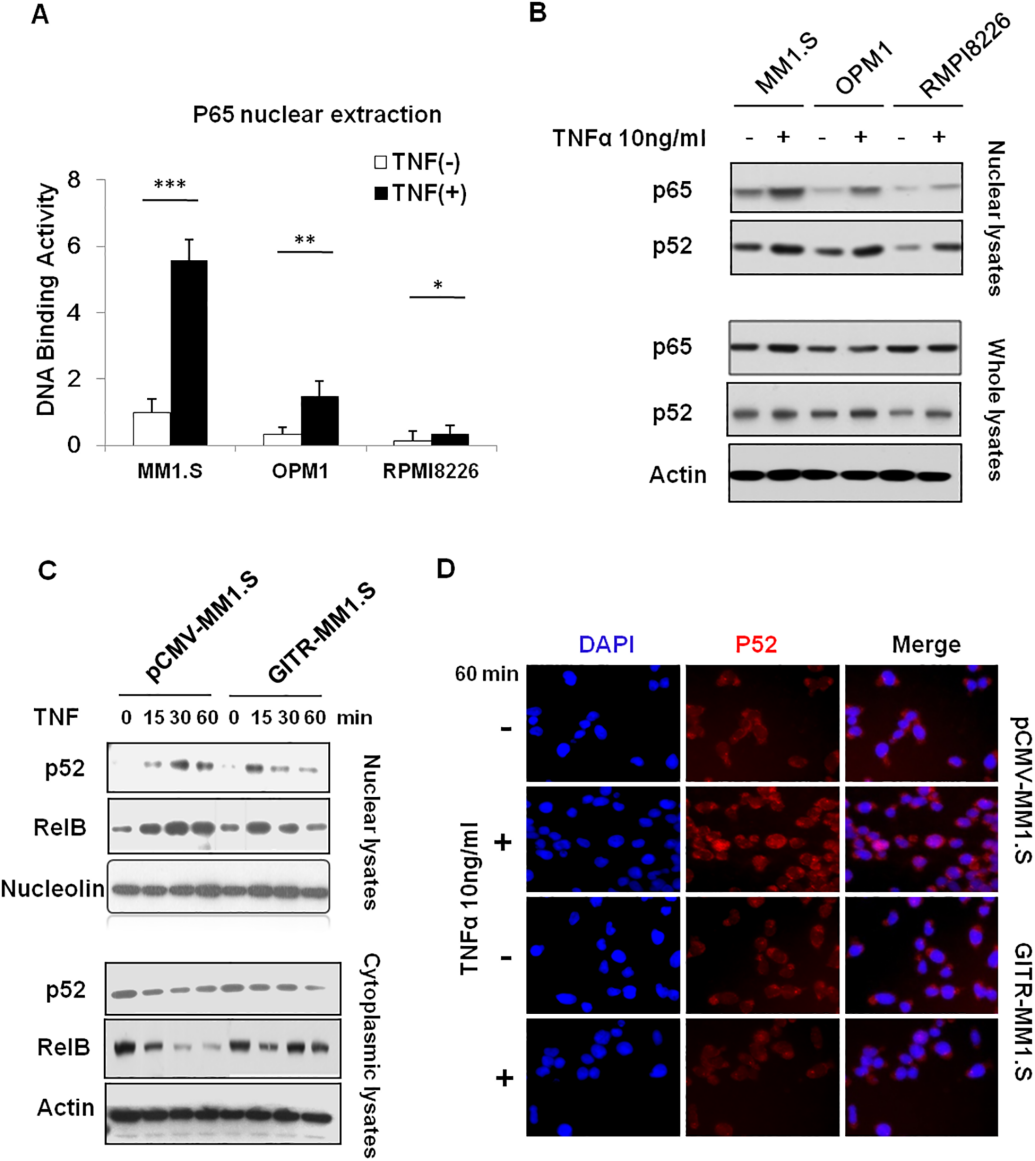
Overexpression of GITR impacts TNFα-induced non-canonical NF-κB activation. A. MM1.S, OPM1 (GITR low) and RPMI8226 (GITR high) cells were exposed to TNFα (10ng/ml) for 60 minutes. NF-κB activity was evaluated by DNA binding ELISA assay. NF-κB p65 transcription factor binding to its consensus sequence on the plate-bound oligo nucleotide was examined from nuclear extracts. Data represent mean ± SD of triplicate experiments. *P<0.05, **P<0.01 and ***P<0.001 compared with indicated groups. B. MM1.S, OPM1 and RPMI8226 cells were exposed to TNFα (10ng/ml) for 60 minutes. Nuclear and whole cell lysates were subjected to western blot using anti-p65, -p52 and Actin antibodies. C. Cells were exposed to TNFα (10ng/ml) for 15, 30, or 60 minutes. Nuclear protein and cytoplasmic extraction were subjected to western blot using anti-p52, -RelB and -nucleolin antibodies. D. pCMV-GITR and GITR-MM1.S cells were harvested at 24 hours after treatment with and without TNF-α (10ng/ml) for 60 minutes. Immunocytochemical analysis was assessed using anti-phospho-NF-κB-p52 antibody, with DAPI used to stain nuclei.

Previous study showed that overexpression of GITR could block canonical NF-κB activation [10]. Herein, to further determine the association of NF-κB activity with GITR expression, we examined NF-κB activation in response to TNFα in three MM cell lines with differential levels of GITR by DNA binding ELISA assay. As shown in Fig 2A, MM1.S and OPM1 cells with low GITR expression showed strong p65 activation. However, there was very weak NF-κB activation observed in RPMI8226 cells, which express high level of GITR. In addition, using western blot assay, we also found that GITR expression impacted p52 nuclear translocation in stimulation with TNFα (Fig 2B). These results suggest that GITR expression negatively correlate with both canonical and non-canonical NF-κB activation in MM cells. To further determine the effect of GITR expression on non-canonical NF-κB pathway, we overexpressed GITR in MM1.S, a GITR-deficient cell line, and found that overexpression of GITR significantly impaired p52/RelB NF-κB translocation into the nuclear in a time-dependent manner (Fig 2C). Furthermore, immunofluorescence staining showed that p52 localization was attenuated upon TNFα stimulation in GITR-overexpressed MM1.S cells (Fig 2D and S1 Fig). Combined with the previous reports, these results suggest that GITR plays an inhibitory role in both canonical and non-canonical NF-κB activation.

### Inhibition of NF-κB activity enhanced TNF-induced apoptosis in GITR-deficient MM cells

**Fig 3:**
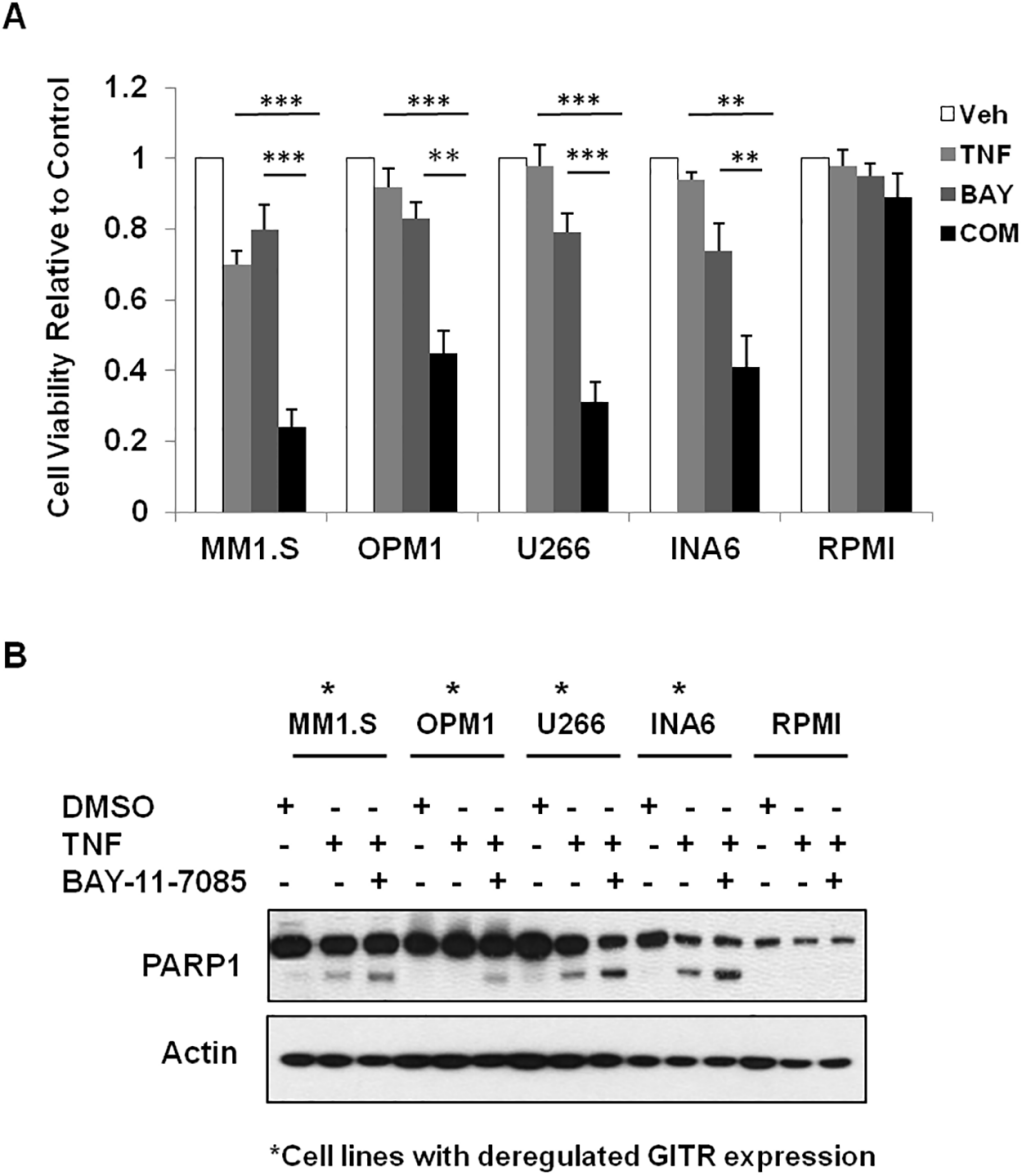
GITR expression correlates with NF-κB activation and sensitivity to NF-κB inhibitor. A. Sensitivity of MM cells to NF-κB inhibitor-BAY-11-7085 was assessed in five MM cell lines. Cell viability was determined by CellTiter-Glo assay. Veh indicating DMSO treated cells. Com indicates combination treatment of TNF with BAY-11-7085. Data represent mean ± SD, **P<0.01 and ***P<0.001 compared with Veh groups. B. MM cell lines were exposed to 10ng/ml TNF with/without BAY-11-7085. Cells were lysed after 12 hours incubation and subjected to Immunoblotting using anti-PARP1 and Actin antibodies.

Previous study showed that loss of GITR resulted in increased NF-κB activity in MM cells [10]. Since increased NF-κB activity has been shown to play an important role in pathogenesis of MM disease, it is very important to determine if loss of GITR mediated NF-κB activation is crucial for MM pathogenesis. To examine this, we performed the cell viability and apoptosis assay in 5 MM cell lines in response to NF-κB inhibitor in the presence of TNFα. We found that NF-κB inhibitor, BAY-11-7085, significantly inhibited cell growth of low GITR expressing cell lines, MM1.S, OPM1, U266 and INA6. In contrast, RPMI cell line, which showed higher GITR level, was not sensitive to BAY-11-7085 in the presence of TNFα (Fig 3A and 3B). These results suggest that loss of GITR mediated NF-κB activation might be important to MM tumorigenesis.

### Expression of GITR is associated with the sensitivity to Bortezomib in MM cells

**Fig 4:**
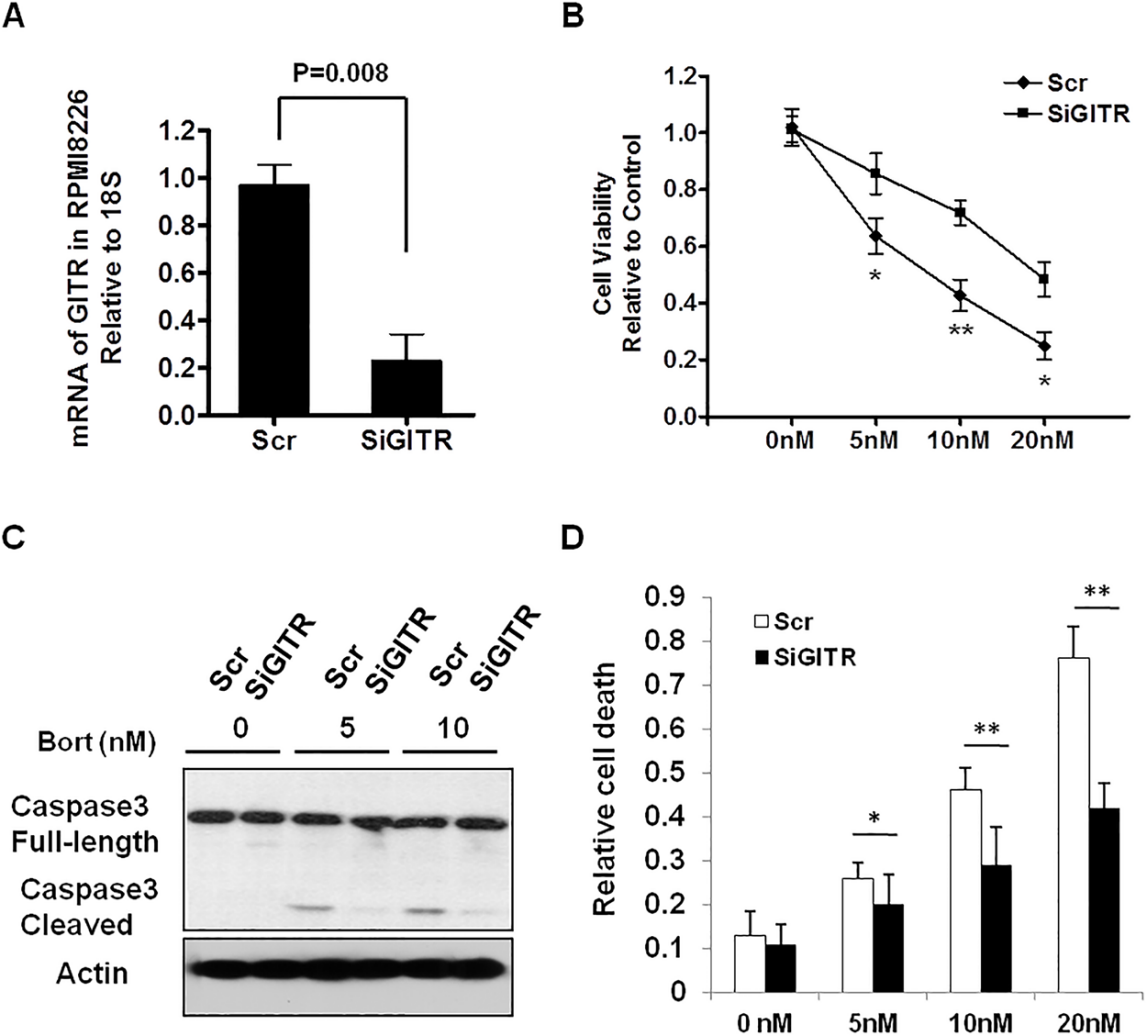
Knockdown of GITR reduced the sensitivity to Bortezomib in RPMI cell line. A. Knockdown efficacy of GITR gene was assessed by real time-PCR. Scr (Scramble) indicates the non-targeting SiRNA control. B. Knockdown of GITR reduced sensitivity to Bortezomib in RPMI cell line. Cell viability was evaluated by CellTiter-Glo assay after 48 hours incubation. Data represent mean ± SD, * P<0.05, and **P<0.01. C. Scr control and SiGITR-transfected RMPI cells were exposed to different doses of Bortezomib and incubated overnight. Cells were lysed and subjected to Immunoblotting using anti-cleaved caspase-3 and Actin antibodies. D. Scr control and SiGITR-transfected RMPI cells were exposed to different doses of Bortezomib and incubated for 24 hours. The number of dead cells were assessed by FACS based PI single staining and quantified by Flowjo software. Data represent mean ± SD, * P<0.05, and **P<0.01 compared with indicated groups.

**Fig 5:**
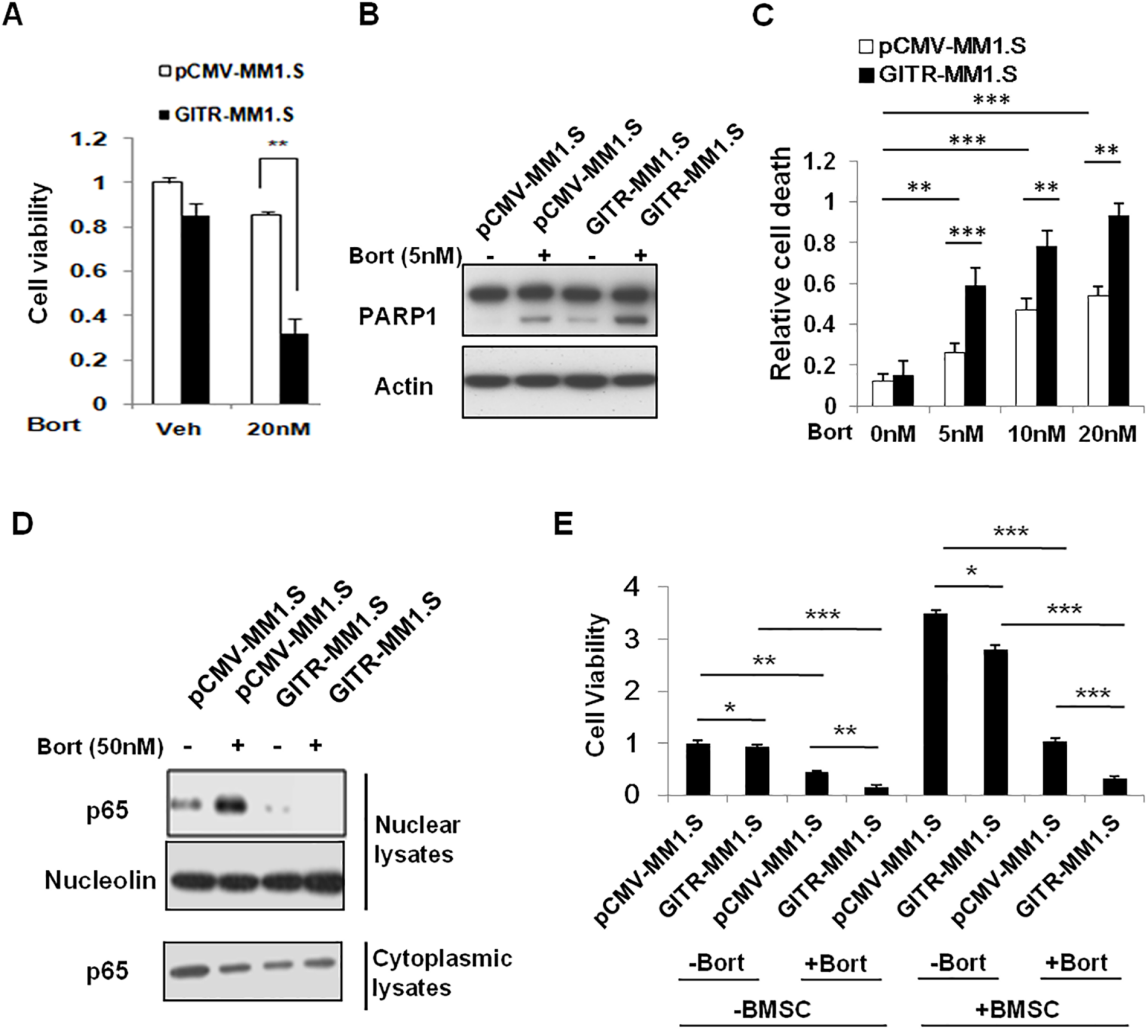
Overexpression of GITR enhanced sensitivity to Bortezomib-induced apoptosis in MM1.S cells. A. Empty vector and GITR-transfected MM1.S cells were treated with Bortezomib for 48 hours. Cell viability was assessed by CellTiter-Glo assay. Data represent mean ± SD, **P<0.01 compared with indicated groups. B. Empty control and GITR expressing MM1.S cells were exposed to different doses of Bortezomib and incubated overnight. Cells were lysed and subjected to Immunoblotting using anti-PARP1 and Actin antibodies. C. Empty control and GITR expressing MM1.S cells were exposed to different doses of Bortezomib and incubated for 24 hours. The number of dead cells were assessed by PI single staining and quantified by Flowjo software. Data represent mean ± SD, **P<0.01 and ***P<0.001 compared with indicated groups. D. NF-κB activity was evaluated in control and GITR expressing MM1.S cells. Nuclear protein lysates were subjected to western blot using anti-p65 and nucleolin antibodies. E. Empty vector and GITR-transfected MM1.S cells were treated with 50nM Bortezomib for 48 hours in co-cultured with or without BMSC. After 48 hours incubation, the cell viability was assessed by CellTiter-Glo assay. Data represent mean ± SD, *P<0.05, **P<0.01 and ***P<0.001 compared with indicated groups.

It has been reported that Bortezomib induces NF-κB activation in MM, we proposed to determine if GITR status can affect MM cells responded to Bortezomib. To explore this, we investigated the sensitivity to Bortezomib by knocking down GITR in RPMI cell line in comparison with control cell line. We found that knockdown of GITR significantly impaired the sensitivity of RPMI to Bortezomib-induced apoptosis (Fig 4B, 4C and 4D). The knockdown efficiency was confirmed by real time PCR, showing mRNA of GITR decreased to about 20% of control cell line (Fig 4A). In contrast, overexpression of GITR in MM1.S, a GITR negative cell line, significantly enhanced Bortezomib-induced apoptosis and reduced MM1.S cell growth in vitro (Fig 5A, 5B and 5C, S2 Fig). Notably, we found that overexpression of GITR inhibited Bortezomib-induced NF-κB activation, supported by decreased NF-κB p65/p50 translocation into the nuclear upon Bortezomib treatment (Fig 5D). Since previous study showed that GITR may also activate p53 pathway, we also overepxressed GITR in U266, a p53 mutated MM cell line, to examine the apoptotic effect of GITR expression in the presence of Bortezomib. Indeed, we found that expression of GITR could also enhance Bortezomib-induced apoptosis in GITR-U266 cells, indicating GITR mediated inhibition of NF-κB activation is crucial for sensitivity of MM cells to Bortezomib (S3 Fig). Furthermore, overexpression of GITR also sensitized MM1.S cells to Bortezomib when co-cultured with bone marrow derived stromal cells (BMSC), suggesting GITR may also play an important role in counteracting the BMSC-mediated survival signals in MM microenvironment (Fig 5E).

### Expression of GITR enhances the cytotoxicity effect of Bortezomib in vivo

**Fig 6:**
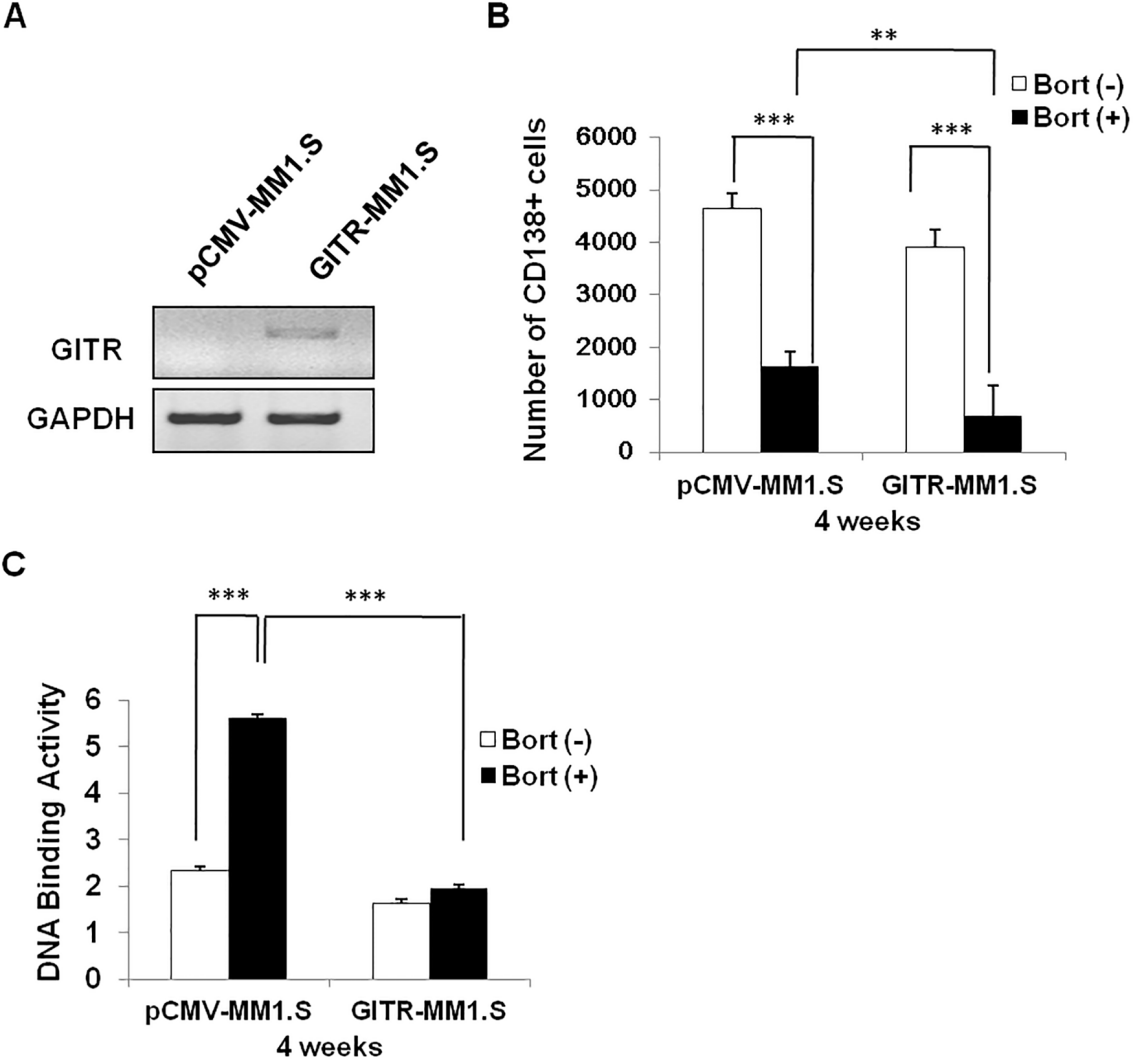
Overexpression of GITR enhanced Bortezomib induced tumor growth inhibition in MM1.S xenograft mice. A. Empty control and GITR expressing cells were isolated from MM1.S xenograft mice and subjected to mRNA extraction. The expression of GITR was examined by real time-PCR. GAPDH was considered as an internal control. B. CD138^+^ human plasma cells were isolated from femur of the four groups of investigated mice. The number of CD138^+^ cell was assessed by flow cytometry. Data represent mean ± SD, **P<0.01 and ***P<0.001 compared with indicated groups. C. NF-κB activity was evaluated by DNA binding ELISA assay. NF-κB p65 transcription factor binding to its consensus sequence on the plate-bound oligo nucleotide was examined from nuclear extracts. Data represent mean ± SD of triplicate experiments. ***P<0.001 compared with indicated groups.

To determine if GITR expression can also have effect on Bortezomib-induced MM tumor growth inhibition in mice model, we assessed the tumor growth upon the treatment of Bortezomib in MM1.S xenograft immune deficiency mice by tail vein injection. The expression of GITR in extracted bone marrow CD138^+^cells was confirmed by quantitative RT-PCR (Fig 6A). Using flow cytometry assay to assess the number of CD138^+^ cells, we found that overexpression of GITR significantly improved the Bortezomib-induced MM tumor inhibition comparing to the control group after 4 weeks treatment with Bortezomib (Fig 6B and S4 Fig). Notably, the number of CD138^+^ plasma cells in the untreated GITR expressing group was less than the controls, suggesting GITR expression had cytotoxicity effect on MM cells. In addition NF-κB activity was also assessed in these isolated bone marrow plasma cells from each group, showing expression of GITR effectively inhibited Bortezomib-induced NF-κB activation in vivo (Fig 6C).

## Discussion

Multiple myeloma is a hematologic cancer, and characterized by uncontrolled plasma cell proliferation in human bone marrow [15]. Bortezomib, one of the most commonly used proteasome inhibitor, has also been approved for the treatment of myeloma [16, 17]. In pre-clinical studies Bortezomib showed a number of different anti-myeloma effects including disruption of the cell cycle and induction of apoptosis [18, 19]. However, only 30–40% relapsed and refractory MM patients were sensitive to Bortezomib in phase II/III clinical trials. In this study, we found that the expression of a TNFRSFs member, GITR, positively correlated with the response to Bortezomib. These observations were verified by both in vitro and in vivo cytotoxicity assay, showing deregulation of GITR could significantly impair the sensitivity to Bortezomib in MM cell lines. In addition, we also showed that the expression of GITR enhanced the cellular response to Bortezomib through inhibiting Bortezomib-induced NF-κB activation. Therefore, we provide a novel molecular mechanism of MM cells resistance to Bortezomib.

Recently, GITR has been identified as a novel tumor suppressor, affecting NF-κB activation in MM. Notably, the expression of GITR significantly correlates with the stage of MM disease, suggesting the role of GITR in MM disease progression [10]. These findings led us to hypothesize that GITR status might be associated with the sensitivity of Bortezomib in MM patients. Indeed, our study showed that GITR played an important role in Bortezomib response, supported by GITR overexpression enhanced the apoptosis in Bortezomib treated MM cells.

Bortezomib treatment can induce NF-κB activation in parallel with I-κB α degradation [20]. The activated NF-κB pathway might affect the sensitivity to Bortezomib treatment in MM patients [21, 22]. Recently, it has been shown that GITR expression impacts NF-κB activation by inhibiting phosphorylation of IKK-beta in MM [10]. Although it is still not clear how GITR modulates NF-κB activity, it raises up a question if loss of GITR influences MM sensitivity to Bortezomib via NF-κB pathway. In fact, by using MM1.S cell line, we found that overexpression of GITR could inhibit Bortezomib-induced NF-κB activation. Furthermore, the MM1.S-GITR xenograft mice also showed decreased NF-κB activity in CD138^+^ plasma cells compared to MM1.S control cells. These findings also suggest that GITR-mediated NF-κB activity is critical for sensitivity to Bortezomib treatment. However, we can’t completely exclude the possibility that p53 activation may also play a role in modulating Bortezomib response since it has been shown that GITR can also lead to more or less p53 activation.

In summary, we report here, that Bortezomib-induced NF-κB activation can be inhibited by overexpression of GITR. Loss of GITR reduced the sensitivity to Bortezomib in MM. Moreover, expression of GITR enhanced the Bortezomib-induced apoptosis in MM cell lines. Therefore, we propose there that GITR status might be used to predict the clinical therapeutic response to Bortezomib in relapsed MM patients.

## Materials and Methods

### Ethics statement

Bone marrow samples from patients with MM and healthy donors were obtained under The Second Hospital of Jilin University IRB approval with written informed consent. In animal studies, mice were treated, monitored, and sacrificed in accordance with approved protocol of The Second Hospital of Jilin University Animal Care and Use Committee.

### Cultured cell lines and primary tumor samples

5 human myeloma cell lines, MM1.S,RPMI, U266(ATCC, Manassas, VA), INA6 and OPM1 (provided by Dr. Y. Zhang, National institute of Health, Bethesda, MD) and human bone marrow stromal cell line (Lonza, Walkersville, MD) were used in this study. All human MM cell lines were cultured as described previously [23]. Primary CD138^+^ MM cells were obtained from BM samples of MM patients (n = 16). CD138^+^ Plasma cells were obtained by using CD138^+^ microbeads selection (Miltenyi Biotec, Auburn, CA). Similarly, CD138^+^ plasma cells were isolated from the BM of 20 healthy donors and pooled together as normal controls.

### Real time-PCR and siRNA transfection

Real time-PCR for GITR was performed on an Applied Biosystems AB7500 Real Time PCR system. All PCR reactions were run in triplicate, and GITR expression relative to 18s was calculated using the 2^–ΔΔCt^ method. The delivery of siRNA into RPMI cells was performed using Lipofectamine 2000 reagent (Invitrogen). Cells were transfected with siRNAs at a final concentration of 50nM. The siRNAs used in this study were: Scrambled ON-TARGETplus nontargeting pool (Dharmacon #D001810-10), and SMARTpool ON-TARGETplus GITR siRNA (Dharmacon #L-006449-00-0005).

### PI staining for cell death assessment

For cell death analysis by flow cytometry, cells were seeded in a 6 cm^2^ dish at 60% confluency. The cells were concomitantly treated with Bortezomib overnight and stained with 10ng/ml propidium iodide (PI). The number of dead cells was determined using a FACS caliber flow cytometer (Becton Dickinson, Oxford, UK) and quantified by FlowJo7.6.5 software.

### Immunofluorescence staining

Immunocytochemical analysis was performed according to the protocol from Cell Signaling Technology Company. In brief, cells were fixed with 16% formaldehyde in phosphate-buffered saline and permeabilized with 0.3% Triton X-100. Cells were blocked in 1X PBS/5% normal goat serum PBS and incubated with primary antibody in 1X PBS/1% BSA/0.3% Triton X-100 at 4°C overnight. In the next day, cells were washed by PBS for three times and incubated with Alexa Fluor 647 goat anti-rabbit secondary antibody (CAT# A-21245, Life Technology) for 2hours. Finally the cell staining was observed under fluorescence microscopy.

### Immunoblotting

Immunoblotting was carried out using standard techniques. Briefly, cells were lysed in ice-cold 1x RIPA lysis buffer and protein concentrations determined. Aliquots (50μg) of protein were denatured in Laemmli loading buffer and separated on precast 4–10% NuPAGE Novex 4–12% Bis-Tris Protein Gels, (Novex-Invitrogen). The antibodies used for immunoblotting included anti—caspase3, p65, p50, Parp-1 RelB (Cell Signaling Technology, Danvers, MA), and Actin (Santa Cruz Biotechnology, Santa Cruz, CA). Nuclear extracts of the cells were prepared using the Nuclear Extraction Kit (Panomics, Redwood City, CA) and subjected to immunoblotting with anti-p65, -p50, (Cell Signaling Technology), and anti-nucleolin (Santa Cruz Biotechnology) antibodies.

### Analysis of NF-κB activity

NF-κB activity was assessed by Active Motif TransAM NF-κB Family Kit, (Active Motif North America, Carlsbad, CA). Briefly, MM.1S cells (control MM1.S and GITR-transfected MM1.S) were treated with Bortezomib. NF-kB-p65 binding to the related DNA sequence on the oligonucleotide coated-plates was studied from nuclear extracts, following the manufacturer’s procedure. For analysis of NF-κB activity in xenograft mice, the whole bone marrow, including femur, skull and vertebrae were crashed and filtered into single cell suspension. To obtain the pure CD138^+^ cell fraction, these cells were isolated by CD138 microbeads selection (Miltenyi Biotec, Auburn, CA).

### Cell viability assay

For cell viability assays, 3000 cells were plated in sterile 96-well plates and cultured overnight. Compounds were then added in serial dilutions. The Bay 11–7084 was purchased from Santa Cruz (CAT# sc-202490). Cellular viability was determined after 48 hours incubation by the CellTiter-Glo Luminescent Cell Viability Assay (Promega). Plates were measured on a THERMO max microplate reader. For stromal co-culture cell assay, BMSCs were obtained from bone marrow of MM patients. Briefly, bone marrow aspirates were subjected to Ficoll-Paque gradient centrifugation (GE Healthcare, Little Chalfont, United Kingdom), and mononuclear cells (MNCs) were collected and expanded in human complete MesenCult medium (STEMCELL Technologies, Canada) for 2 weeks. Then a confluent monolayer was generated by plating 10×10^5^ BMSCs in a 96-well plate for additional 48 hours. MM.1S cells were treated with Bortezomib and plated in co-culture with BMSCs for 48 hours at 37°C. Cell Viability was assessed by the CellTiter-Glo Luminescent intensity.

### In vivo tumor progression of MM cells

5 SCID mice for each group were injected with 5X10^6^ empty control MM1.S and GITR-MM1.S cells. The treatments for each group were as follows: (1) No treatment (control), (2) Bortezomib, 1 mg/kg body weight on 7, 10, 13, 17, 20, and 24 days after MM1.S cells injection. Body weight and the number of tumor cells were measured at least twice a week. All the mice were sacrificed by CO_2_ asphyxiation after 24 days treatment with/without Bortezomib. Bone marrow tissue of femur as well as peripheral blood was isolated from the four groups. Mice bone marrow tissues were crushed and filtered into single cell suspensions. Anti human CD138 monoclonal antibody was used to examine MM cells growth by flow cytometry as previously described [10].

### Statistical analysis

The statistical significance between groups was assessed with Student’s *t*-test. Experiments were repeated in triplicates. A value of P<0.05 was considered statistically significant and indicated by one asterisks. P <0.01or <0.001 was represented by two asterisks or three asterisks respectively. Error bars shown in the figures represent standard deviations. Statistical analyses were carried out using Microsoft Excel software.

## Supporting Information

S1 Fig Overexpression of GITR inhibits TNFα-induced p52 nuclear translocation. pCMV-GITR and GITR-MM1.S cells were harvested at 24 hours after treatment with 10ng/ml TNF-α for 60 minutes. Immunocytochemical analysis was assessed using anti-phospho-NF-κB-p52 antibody and DAPI for nuclei staining. Representative images are shown with higher magnification.
(TIF)

S2 Fig Overexpression of GITR enhances Bortezomib-induced cell death inMM1.S cell line. Empty control and GITR expressing MM1.S cells were exposed to different doses of Bortezomib and incubated for 24 hours. The dead cells were assessed by PI single staining. Data are shown as representative dot plot of flow cytometry analysis in Fig 5C.
(TIF)

S3 Fig Overexpression of GITR enhances Bortezomib-induced apoptosis in U266 cell line. Empty control and GITR expressing U266 cells were exposed to 5nM Bortezomib and incubated overnight. Cells were lysed and subjected to Immunoblotting using anti-PARP1, IκB, Myc and Actin antibodies. The expression of GITR enhanced Bortezomib-induced apoptosis in GITR-U266 cells, indicating GITR mediated inhibition of NF-κB activation is crucial for sensitivity of MM cells to Bortezomib.
(TIF)

S4 Fig Expression of GITR enhances the cytotoxicity effect of Bortezomib in xenograft mice. CD138^+^ human plasma cells were isolated from femur of the four groups of investigated mice. Data represent the dot plot of flow cytometry analysis in Fig 6B.
(TIF)
