# Supplementary figures and images for: Expression of GITR Enhances Multiple Myeloma Cell Sensitivity to Bortezomib
Source: PLoS One. 2015 May 14;10(5):e0127334. doi: 10.1371/journal.pone.0127334 (PMC4431824; doi:10.1371/journal.pone.0127334)

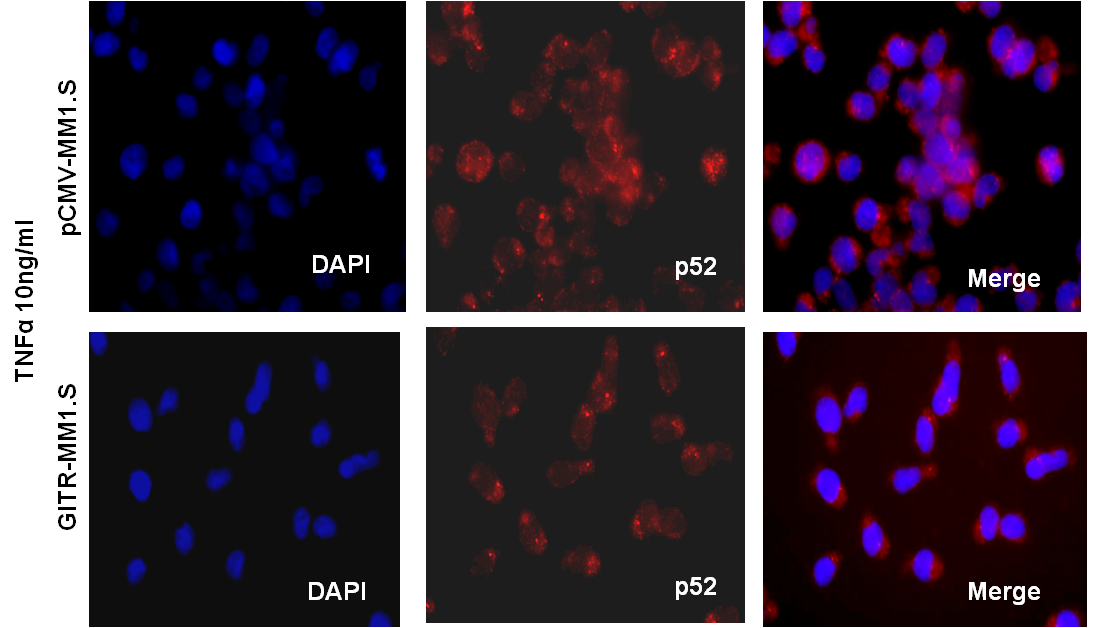

Supplement: S1 Fig — pCMV-GITR and GITR-MM1.S cells were harvested at 24 hours after treatment with 10ng/ml TNF-α for 60 minutes. Immunocytochemical analysis was assessed using anti-phospho-NF-κB-p52 antibody and DAPI for nuclei staining. Representative images are shown with higher magnification. (TIF) [file pone.0127334.s001.tif]

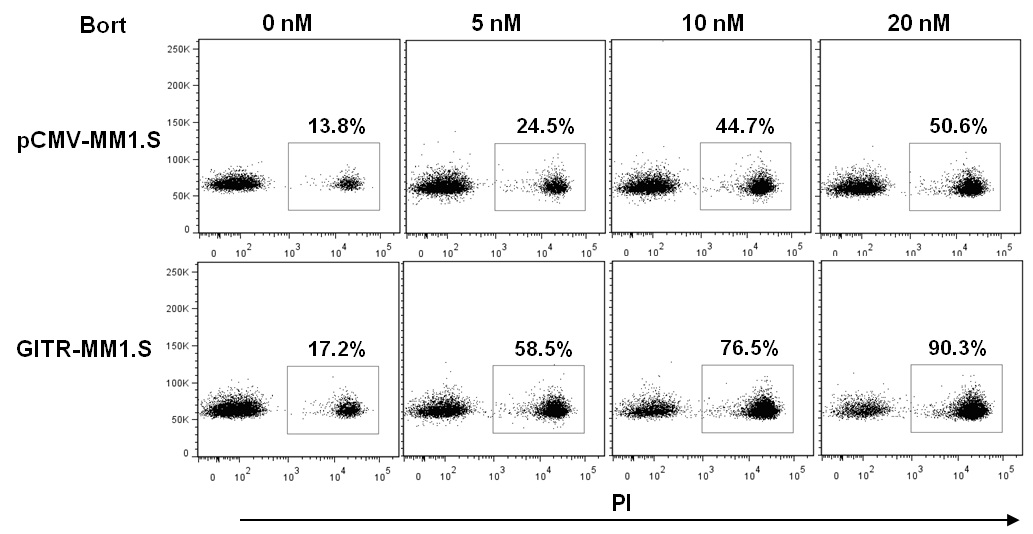

Supplement: S2 Fig — Empty control and GITR expressing MM1.S cells were exposed to different doses of Bortezomib and incubated for 24 hours. The dead cells were assessed by PI single staining. Data are shown as representative dot plot of flow cytometry analysis in Fig 5C. (TIF) [file pone.0127334.s002.tif]

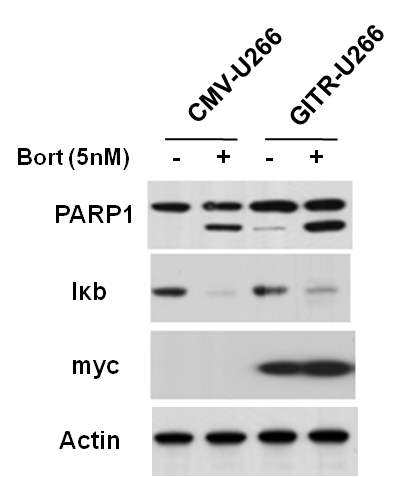

Supplement: S3 Fig — Empty control and GITR expressing U266 cells were exposed to 5nM Bortezomib and incubated overnight. Cells were lysed and subjected to Immunoblotting using anti-PARP1, IκB, Myc and Actin antibodies. The expression of GITR enhanced Bortezomib-induced apoptosis in GITR-U266 cells, indicating GITR mediated inhibition of NF-κB activation is crucial for sensitivity of MM cells to Bortezomib. (TIF) [file pone.0127334.s003.tif]

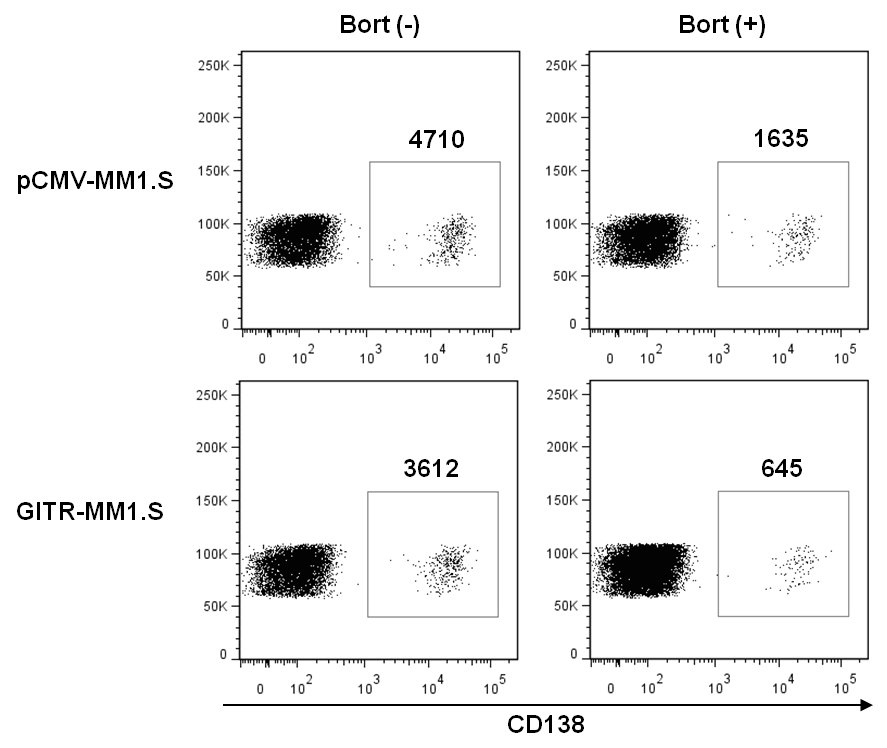

Supplement: S4 Fig — CD138+ human plasma cells were isolated from femur of the four groups of investigated mice. Data represent the dot plot of flow cytometry analysis in Fig 6B. (TIF) [file pone.0127334.s004.tif]
